# Transcriptional Responses and Gentiopicroside Biosynthesis in Methyl Jasmonate-Treated *Gentiana macrophylla* Seedlings

**DOI:** 10.1371/journal.pone.0166493

**Published:** 2016-11-16

**Authors:** Xiaoyan Cao, Xiaorong Guo, Xinbing Yang, Huaiqin Wang, Wenping Hua, Yihan He, Jiefang Kang, Zhezhi Wang

**Affiliations:** 1 Key Laboratory of the Ministry of Education for Medicinal Resources and Natural Pharmaceutical Chemistry, National Engineering Laboratory for Resource Development of Endangered Crude Drugs in Northwest of China, College of Life Sciences, Shaanxi Normal University, Xi’an, China; 2 Department of Biological Science and Technology, Shaanxi XueQian Normal University, Xi’an, China; East Carolina University, UNITED STATES

## Abstract

*Gentiana macrophylla*, a medicinal plant with significant pharmacological properties, contains the bioactive compound gentiopicroside. Methyl jasmonate (MeJA) is an effective elicitor for enhancing the production of such compounds. However, little is known about MeJA-mediated biosynthesis of gentiopicroside. We investigated this phenomenon as well as gene expression profiles to determine the molecular mechanisms for MeJA-mediated gentiopicroside biosynthesis and regulation in *G*. *macrophylla*. Our HPLC results showed that *Gentiana macrophylla* seedlings exposed to MeJA had significantly higher concentrations of gentiopicroside when compared with control plants. We used RNA sequencing to compare transcriptional profiles in seedlings treated for 5 d with either 0 μmol L^-1^ MeJA (C) or 250 μmol L^-1^ MeJA (M5) and detected differentially expressed genes (DEGs). In total, 77,482 unique sequences were obtained from approximately 34 million reads. Of these, 48,466 (57.46%) sequences were annotated based on BLASTs performed against public databases. We identified 5,206 DEGs between the C and M5 samples, including genes related to the α-lenolenic acid degradation pathway, JA signaling pathway, and gentiopicroside biosynthesis. Expression of numerous enzyme genes in the glycolysis pathway was significantly up-regulated. Many genes encoding transcription factors (e.g. ERF, bHLH, MYB, and WRKY) also responded to MeJA elicitation. Rapid acceleration of the glycolysis pathway that supplies precursors for IPP biosynthesis and up-regulates the expression of enzyme genes in that IPP pathway are probably most responsible for MeJA stimulation of gentiopicroside synthesis. Our qRT-PCR results showed that the expression profiles of 12 gentiopicroside biosynthesis genes were consistent with the RNA-Seq data. These results increase our understanding about how the gentiopicroside biosynthesis pathway in *G*. *macrophylla* responds to MeJA.

## Introduction

The plant hormone methyl jasmonate (MeJA) is an efficient elicitor of secondary metabolite production [1]. Those metabolites include flavonoids, phenolic and polyphenolic compounds, terpenoids, and alkaloids. Such bioactive compounds in plants represent valuable and unique resources for food additives, cosmetics, and pharmaceutical drugs [2]. Treating plants with MeJA can trigger the biosynthesis of terpenoids, alkaloids, phenylpropanoids, and phytoalexins through extensive transcriptional reprogramming of their metabolism [1, 3–7].

*Gentiana macrophylla* Pall (family Gentianaceae) is a perennial medicinal plant prescribed in China since ancient times to treat arthralgia, stroke, hemiplegia, pain, jaundice, infantile malnutrition, and osteoarthritis [8]. Its dried roots are officially listed in the *Chinese Pharmacopoeia* under the name Radix Gentianae Macrophyllae (Qin-jiao in Chinese) and are frequently used to dispel rheumatism and ease pain [9]. Gentiopicroside, an abundant and indicative ingredient in Qin-jiao, is the most important active component of total secoiridoid glycosides and has significant anti-inflammatory, analgesic, and antibacterial properties, as well as biological activity for treating osteoarthritis and strengthening gastric motility [10,11]. Although levels of gentiopicroside in Qin-jiao are influenced by soil elements and fertilization [12,13], the effect of MeJA in regulating those concentration has not been investigated.

Gentiopicroside is synthesized via the secoiridoid pathway. This pathway has been well studied and reviewed in *Catharanthus roseus* [14,15], and most steps have been identified. In the first step within higher plants, isopentenyl diphosphate (IPP), a precursor of terpenoids, is formed through either the plastidial 2-C-methyl-D-erythritol-4-phosphate (MEP) pathway or the cytosolic mevalonic acid (MVA) pathway. The allylic isomer of IPP, dimethylallyl diphosphate, reacts with one IPP in a head-to-tail fashion to form geranyl diphosphate, which is then catalyzed and converted into geraniol. The secoiridoid pathway starts with geraniol and proceeds through a series of reaction steps leading to the formation of secologanin [15]. Ultimately, secologanin is converted into gentiopicroside and other secoiridoids through several currently unknown steps [16].

In contrast to conventional methods, such as single gene cloning and DNA microarrays, that yield a limited amount of genetic information, RNA-seq is powerful tool for analyzing differential gene expression with high resolution at the whole-genome level [17,18]. In particular, transcriptome analysis can reveal relationships between plant gene expression and phenotype [19–21]. No previous regulatory mechanisms for gentiopicroside biosynthesis have been reported for *G*. *macrophylla*. However, *de novo* analysis using next-generation sequencing technologies can provide a robust platform for elucidating the mechanisms that might influence the accumulation of gentiopicroside in that species.

Because data are lacking for the means by which gentiopicroside production in *G*. *macrophylla* is modulated by MeJA, we monitored concentrations of that compound in MeJA-treated seedlings. Methyl jasmonate stimulates gentiopicroside biosynthesis. Therefore, RNA-seq can be used to analyze differential gene expression over time in MeJA-treated plants versus the control. In general, applications of MeJA trigger profound transcriptional reprogramming in plant cells to manipulate the machinery that controls a wide range of metabolite biosynthesis via interplay of both positive and negative regulators [22]. Improving our understanding of the events between MeJA application and gentiopicroside accumulation will be useful for developing strategies to enhance production in *G*. *macrophylla*. Therefore, our objective here was to identify the relevant metabolic pathways for major MeJA-responsive genes and decipher the molecular mechanism by which MeJA stimulates yields.

## Materials and Methods

### Plant growth conditions and treatments

Seeds of *Gentiana macrophylla* were collected from the cultivation base of our lab, which is located in Taibai county, Shaanxi province, China. They were surface-sterilized with 2% sodium hypochlorite for 9 min, followed by five rinses with distilled water. After being kept in the dark for 2 d, they were germinated on culture dishes containing an MS solid medium (16-h photoperiod, 20°C). One-month-old seedlings were transferred to either a fresh MS solid medium (control; 0 μmol L^-1^ MeJA) or an MS medium supplemented with 250 μmol L^-1^ MeJA. Whole seedling samples were collected after 1, 3, 5, 7, 9, and 11 d of treatment and dried to constant weight at 40°C for the determination of gentiopicroside contents. The experiments were performed in three individual biological replications and every treatment contained more than 30 seedlings.

### Measurements of gentiopicroside

Gentiopicroside concentrations were determined via High Performance Liquid Chromatography (HPLC). All sample solutions and stock solutions of gentiopicroside were prepared as we have described before [23]. Chromatographic separations were conducted with a C18 column (250 × 4.6 mm, 5 μm particle size; Agilent Technologies Inc., USA) on an Agilent 1260 Infinity LC system, using a solvent system comprising 70% ddH_2_O (A) and 30% methanol (B). The flow rate was adjusted to 0.8 mL min^-1^ and the detection wavelength was 245 nm. All separations were performed at 25°C.

### RNA isolation

Total RNA was isolated using TRIzol^®^ Reagent (Invitrogen, USA) according to the manufacturer’s protocol. Quality of the RNA was assessed on agarose gels and the concentration was determined with a NanoDrop ND2000 Spectrophotometer (NanoDrop Technologies Inc., USA).

### cDNA library construction and sequencing

Two cDNA libraries—C (control) and M5 (MeJA treatment for 5 d)—were generated using mRNA-Seq Sample Prep Kits (Illumina, USA) according to the manufacturer’s instructions. Magnetic beads containing poly-T molecules were used to isolate the poly(A) mRNA from 20 μg of total RNA. Following purification, the samples were fragmented into small pieces using divalent cations at 94°C for 5 min, then converted into first- and second-strand cDNA with a SuperScript double-stranded cDNA synthesis kit (Invitrogen). The synthesized cDNA was subject to end repair and adenylation of the 3' ends and purified using a QIAquick PCR Purification Kit (QIAGEN, Germany). Afterward, Illumina paired-end adapters were ligated to the resulting cDNA fragments. Each cDNA library was constructed with an insert size of 200 bp. After quality was verified on an Agilent 2100 Bioanalyzer, deep-sequencing was performed with an Illumina HiSeq4000. In all, 150 bp paired-end reads were generated.

### *De novo* assembly and gene annotation

Raw reads were filtered by the Illumina pipeline prior to assembly. We removed any reads that showed adapter contamination or for which more than 20% of the bases had quality values ≤10 or more than 5% were unknown nucleotides. The high quality clean reads were then randomly clipped into overlapping K-mers with default K = 25 for assembly with the Trinity, a short-reads assembling program [24]. The resulting sequences, termed unigenes, from each sample’s assembly were further processed for sequence-splicing and removal of all redundancy to acquire non-redundant unigenes that were as long as possible. Finally, Blast X alignments were made (E-values <0.00001) between the unigenes and protein databases NR, SwissProt, Pfam, KEGG, KOG, and COG. Sequence directions were decided and functional annotations were assigned for the unigenes based on the best alignment results. Combining NR annotation with the Blast2GO program (v2.5.0; release 20120801) [25], we obtained GO annotations for the unigenes. All GO functional classifications was produced by WEGO software [26] and pathway assignments were performed in conjunction with the KEGG database [27].

### Analysis of differentially expressed genes (DEGs)

Normalized expression levels for the unigenes were calculated per the FPKM method [28]. The significance of differential transcript abundance was valued according to the false discovery rate (FDR) [29]. Only those differentially expressed genes (DEGs) with FDRs ≤0.001 and absolute fold-changes ≥2 were reserved. For examining pathway enrichment, all DEGs were mapped to terms in the KEGG database to identify significantly over-represented metabolic pathways or signal transduction pathways. We primarily focused on differentially regulated pathways closely related to the biosynthesis of gentiopicroside.

### Validation of gene expression by qRT-PCR

We selected 12 unigenes involved in gentiopicroside production for qRT-PCR experiments. Gene-specific primer pairs were designed by Primer Premier 5.0 software, and *SAND1* served as the reference gene [30]. Total RNA was isolated from the C and M5 samples using TRIzol^®^ Reagent (Invitrogen) according to the manufacturer’s protocol. After treatment with DNase I (Tiangen, China), 1 μg of RNA was used in reverse-transcription with PrimeScript TM 1st Strand cDNA Synthesis Kits (TaKaRa, Japan). Quantitative reactions were performed on a LightCycler^®^ 96 real-time PCR detection system (Roche, Switzerland), using SYBR_ Premix Ex Taq^™^ (TaKaRa, Japan). Reaction conditions included an initial 95°C for 10 min, then 40 cycles of 95°C for 15 s, followed by 60°C for 25 s. Relative expression levels for each unigene were compared between the two sample types and were calculated by the 2^-ΔΔCt^ method [31]. All data were expressed as means ± SD after normalization. Primer sequences used for qRT-PCR are listed in S8 Table.

### Availability of supporting data

The data set supporting the results presented in this paper is available from the NCBI Sequence Read Archive repository (http://www.ncbi.nlm.nih.gov/sra/) under Accession Number SRP078971.

## Results

### Effects of MeJA on gentiopicroside biosynthesis in *Gentiana macrophylla* seedlings

**Fig 1:**
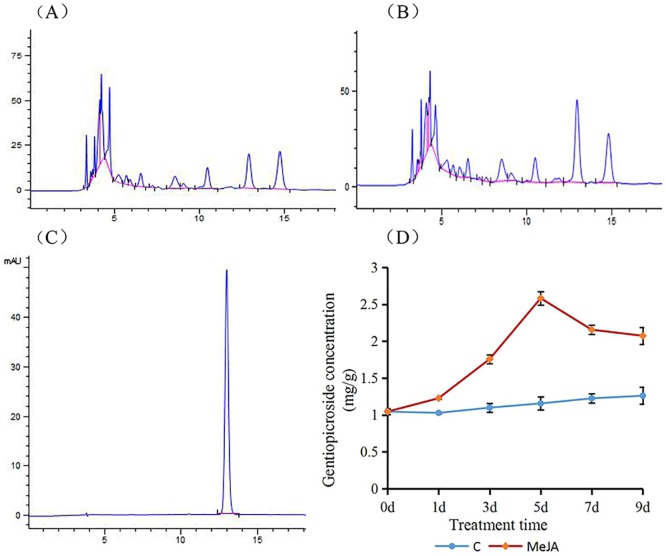
Effects of MeJA application (250 μmol L^-1^) on gentiopicroside biosynthesis in *Gentiana macrophylla* seedlings. (A). Chromatogram of extraction from seedlings not exposed to MeJA. (B). Chromatogram of extraction from seedlings after 5 d of MeJA treatment. (C). Chromatogram of gentiopicroside standard. (D). Changes over time in gentiopicroside concentrations in treated seedlings relative to levels in untreated control (C) plants.

Induction of gentiopicroside biosynthesis in response to MeJA has not been previously described. Here, gentiopicroside production was stimulated in one-month-old *G*. *macrophylla* seedlings treated with 250 μmol L^-1^ MeJA (Fig 1). Our HPLC results showed that levels of gentiopicroside increased to 1.23, 1.76, 2.58, 2.16, and 2.07 mg g^-1^, concentrations that were 19.3, 59.9, 123.3, 75.9, and 64.4% higher than those measured in corresponding control plants (C; 0 μmol L^-1^ MeJA) on Days 1, 3, 5, 7, and 9, respectively, of this experiment. This is the first report to demonstrate that MeJA treatment influences gentiopicroside biosynthesis in this species. We utilized *G*. *macrophylla* seedlings exposed to MeJA for 5 d for RNA-seq analysis because this span of time is associated with the greatest contrast in gentiopicroside concentrations between control (untreated) plants and those to which MeJA has been applied.

### Sequencing and *de novo* assembly

Two cDNA libraries from *G*. *macrophylla* seedlings—C (control) and M5 (MeJA treatment for 5 d)—were sequenced by Illumina deep sequencing to obtain approximately 18.16 and 15.74 million high-quality clean reads for C and M5, respectively. Each read averaged 298 bp long. The Q30 value (percentage of sequences with a sequencing error rate <0.1%) was 94.93% for C and 94.28% for M5. Read lengths were 150 bp×2. Assembling all trimmed reads produced 14,952,699 contigs from the two libraries, which were then joined into unigenes based on the paired-end information. This generated 54,841 (C) and 62,179 (M5) unigenes (total of 77,482 unigenes, including overlaps between C and M5). These had an average length of 764 bp and an N50 of 1,169 bp (i.e., 50% of the assembled bases were incorporated into unigenes at least 1,169 nt long) (Table 1). All unigenes were longer than 200 bp, 46.55% were more than 500 bp long, and 14.56% (12,559) were longer than 1,000 bp.

**Table 1:** Overview of sequencing and assembly.

	C	M5	Total
**Total clean reads**	18,163,693	15,744,104	
**Total clean nucleotides (nt)**	5,409,889,330	4,697,111,888	
**Q30 percentage**	94.93	94.28	
**Unigene**			
**Total number**	54,841	62,179	77,482
**Total length (nt)**	40,033,906	40,520,058	59,196,259
**Mean length (nt)**	692	652	764
**N50**	912	967	1,169

### Functional annotation and classification

We performed function annotation of the generated unigenes by using BLASTX to search reference sequences against results from the NCBI non-redundant protein databases (NR), SwissProt, Protein family (Pfam), euKaryotic Orthologous Groups (KOG), Kyoto Encyclopedia of Genes and Genomes (KEGG), Gene Ontology (GO), and Cluster of Orthologous Groups (COG). A total of 48,466 significant BLAST hits (57.46% of all unigenes) were returned. Among them, 47,172 (97.33%) were found in NR, 31,261 (64.50%) in Swiss-Prot, 31,705 (65.42%) in Pfam, 28,899 (59.63%) in KOG, 18,720 (38.63%) in KEGG, 27,499 (56.74%) in GO, and 15,178 (31.32%) in COG (S1 Table).

**Fig 2:**
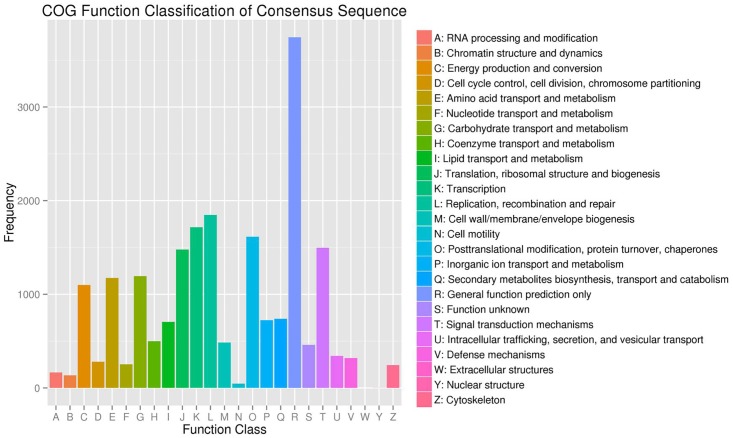
COG classifications of assembled unigenes.

The COG classifications showed that 20,770 assembled unigenes were clustered into 25 functional categories (Fig 2). The largest category was “General function prediction only” (3,744 unigenes; 18.03% of the total), followed by “Replication, recombination and repair” (1,848; 8.90%), “Transcription” (1,716; 8.26%), “Posttranslational modification, protein turnover, chaperones” (1,615; 7.78%), and “Signal transduction mechanisms” (1,498; 7.21%). With only one unigene, “Extracellular structures” was the smallest group while 460 (2.21%) unigenes were annotated as “Function unknown”.

**Fig 3:**
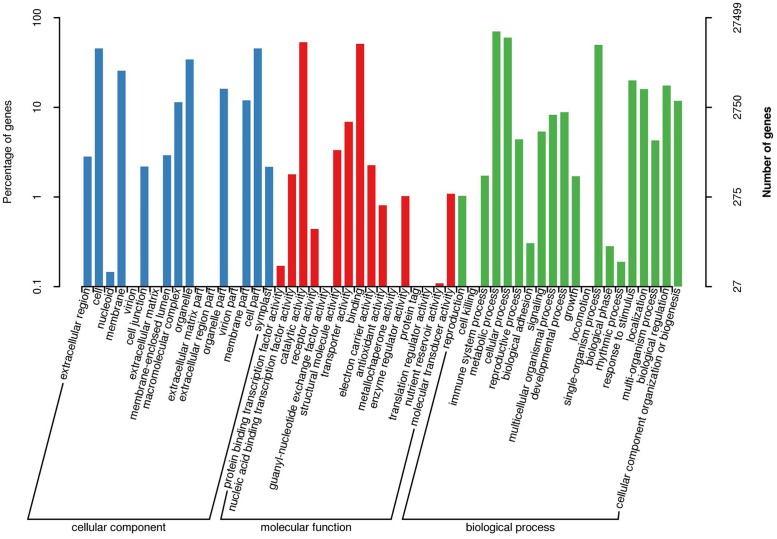
GO classification of assembled unigenes.

We also used GO assignments to classify the predicted functions of 166,401 unigenes into three main categories: “cellular component” (55,286; 33.22%), “molecular function” (33,694; 20.25%), and “biological process” (77,421; 46.53%) (Fig 3). The largest groups within molecular function included unigenes with “catalytic activity” (14,705; 8.84%) and binding activity (14,024; 8.43%). For biological process, the largest unigene groups were “metabolic process” (19,348; 11.62%), “cellular process” (16,456; 9.89%), “single-organism process” (13,653; 8.20%), “response to stimulus” (5,476; 3.29%), and “biological regulation” (4,803; 2.89%).

### Analysis of KEGG pathways and differentially expressed genes (DEGs)

We conducted a KEGG pathway-based analysis to obtain a better understanding of the biological functions of these unigenes. Among the 18,720 annotated transcripts mapped to 128 KEGG pathways, 20 of the most-represented pathways are shown in S2 Table. In all, 2,443 unigenes were assigned to 25 secondary-metabolite pathways (Table 2).

**Table 2:** Secondary metabolism pathways in *Gentiana macrophylla* sequencing.

Pathway	Pathway ID	Number of unigenes
Glycolysis / Gluconeogenesis	ko00010	375
Phenylpropanoid biosynthesis	ko00940	349
Amino sugar and nucleotide sugar metabolism	ko00520	329
Purine metabolism	ko00230	320
Citrate cycle (TCA cycle)	ko00020	205
Pentose phosphate pathway	ko00030	178
Terpenoid backbone biosynthesis	ko00900	161
Steroid biosynthesis	ko00100	81
Carotenoid biosynthesis	ko00906	68
Flavonoid biosynthesis	ko00941	58
Tropane, piperidine, and pyridine alkaloid biosynthesis	ko00960	52
Sesquiterpenoid and triterpenoid biosynthesis	ko00909	46
Isoquinoline alkaloid biosynthesis	ko00950	41
Stilbenoid, diarylheptanoid, and gingerol biosynthesis	ko00945	38
Diterpenoid biosynthesis	ko00904	35
Zeatin biosynthesis	ko00908	24
Limonene and pinene degradation	ko00903	23
Brassinosteroid biosynthesis	ko00905	20
Monoterpenoid biosynthesis	ko00902	19
Caffeine metabolism	ko00232	10
Anthocyanin biosynthesis	ko00942	4
Flavone and flavonol biosynthesis	ko00944	4
Carbapenem biosynthesis	ko00332	1
Isoflavonoid biosynthesis	ko00943	1
Betalain biosynthesis	ko00965	1

The Fragments Per Kb per Million reads (FPKM) method was used to calculate unigene expression levels and, ultimately, identify DEGs. When M5 and C treatments were compared, 3,805 unigenes were up-regulated and 1401 were down-regulated in response to MeJA applications. All DEGs are shown in S3 Table while the 20 unigenes most up- or down-regulated between M5 and C samples are presented in S4 Table.

**Fig 4:**
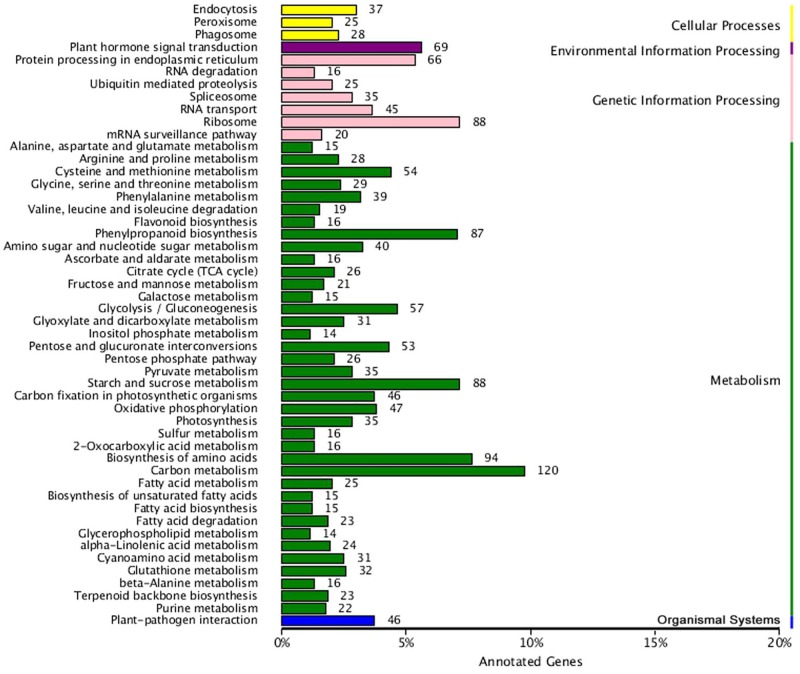
KEGG pathway enrichment analysis of DEGs.

Our KEGG analysis enabled us to identify 38 significantly enriched metabolic pathways or signal transduction pathways for those DEGs (Fig 4). To examine the molecular basis for jasmonate (JA) stimulation of gentiopicroside production in *G*. *macrophylla*, we focused on glycolysis, the IPP biosynthesis pathway, α-linolenic acid metabolism, plant hormone signal transduction, and related transcription factors (TFs).

### DEGs involved in JA biosynthesis and the JA signaling pathway

**Fig 5:**
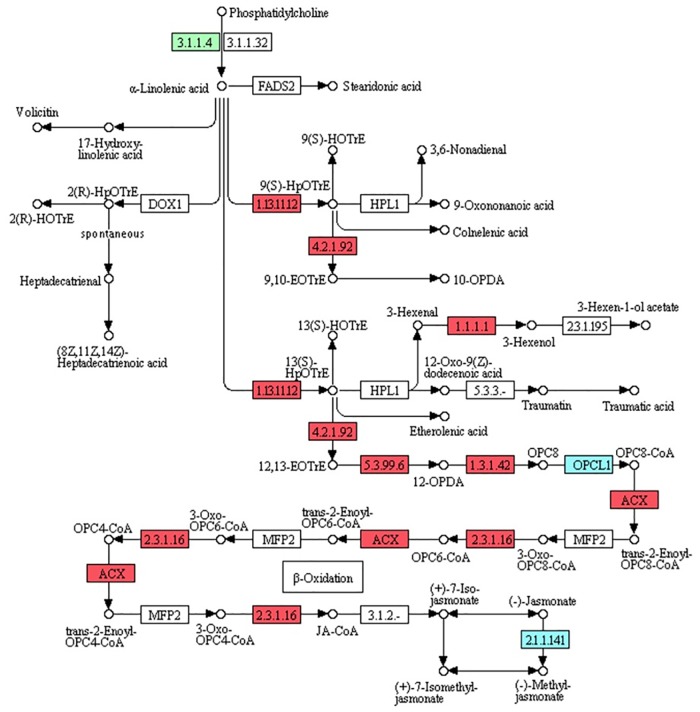
DEGs involved in alpha-linolenic acid metabolism in M5 samples compared with C samples. 3.1.1.4, secretory phospholipase A2; 1.13.11.12, lipoxygenase; 4.2.1.92, allene oxide synthase; 1.1.1.1, alcohol dehydrogenase; 5.3.99.6, allene oxide cyclase; 1.3.1.42, 12-oxophytodienoic acid reductase; OPCL1, OPC-8:0 CoA ligase 1; ACX, acyl-CoA oxidase; 2.3.1.16, acetyl-CoA acyltransferase 1; 2.1.1.141, jasmonate O-methyltransferase. Green box, putative encoding gene is down-regulated; red boxes, putative encoding genes are up-regulated; blue boxes, some putative encoding genes are up-regulated while others are down-regulated.

As an elicitor of the production of bioactive metabolites, JA triggers a transcriptional reprogramming of plant metabolism, resulting in a concerted upregulation of expression for genes that encode enzymes involved in specific, specialized metabolic pathways [32]. We investigated changes in the expression of genes closely associated with α-linolenic acid metabolism, which finally leads to JA biosynthesis, and were interested to find 22 DEGs (Table 3; Fig 5). Of those, 19 DEGs encoding putative lipoxygenase (LOX), allene oxide synthase (AOS), allene oxide cyclase (AOC), 12-oxophytodienoic acid reductase (OPR), OPC-8:0 CoA ligase 1 (OPCL1), acyl-CoA oxidase (ACOX), acetyl-CoA acyltransferase 1 (ACAA1), and jasmonate O-methyltransferase (JMT) showed increased transcript abundance (Table 3; Fig 5).

**Table 3:** DEGs up-regulated in JA biosynthesis and JA signaling pathway in M5 samples compared with expression in C samples.

Enzyme (ID)	KEGG orthology	Sequence ID	log2FC	Fold-change
LOX (EC:1.13.11.12)	K00454	c63841.graph_c0	2.32	4.99
		c70365.graph_c0	6.17	72.00
		c75434.graph_c0	5.55	46.85
		c82675.graph_c0	3.03	8.17
		c82675.graph_c1	2.67	6.36
		c82963.graph_c0	6.83	113.77
AOS (EC:4.2.1.92)	K01723	c66622.graph_c0	4.02	16.22
AOC (EC:5.3.99.6)	K10525	c62754.graph_c0	1.96	3.89
OPR (EC:1.3.1.42)	K05894	c78466.graph_c2	1.96	3.89
OPCL1 (EC:6.2.1.-)	K10526	c75067.graph_c0	2.10	4.29
		c85214.graph_c0	6.01	64.45
		c58704.graph_c0	-4.46	22.01
ACX (EC:1.3.3.6)	K00232	c16978.graph_c0	4.02	16.22
		c64688.graph_c0	5.70	51.98
ACAA1 (EC:2.3.1.16)	K07513	c52442.graph_c0	4.02	16.221
		c71568.graph_c0	5.80	55.72
		c84427.graph_c0	7.80	222.86
JMT (EC:2.1.1.141)	K08241	c49619.graph_c0	1.60	3.03
		c55315.graph_c0	4.26	19.16
		c82386.graph_c0	2.79	6.92
		c69211.graph_c0	-3.03	8.17
		c71815.graph_c0	-1.71	3.27
JAZ protein	K13464	c37174.graph_c0	4.27	19.29
		c38617.graph_c0	4.72	26.35
		c48886.graph_c0	5.09	34.06
		c63000.graph_c0	2.77	6.82
		c65729.graph_c0	4.02	16.22
		c66883.graph_c0	1.88	3.68
		c71121.graph_c0	4.86	29.04
		c86508.graph_c0	4.42	21.41
MYC2	K13422	c77634.graph_c0	2.11	4.32
		c78484.graph_c0	1.75	3.36

### DEGs involved in gentiopicroside biosynthesis

**Fig 6:**
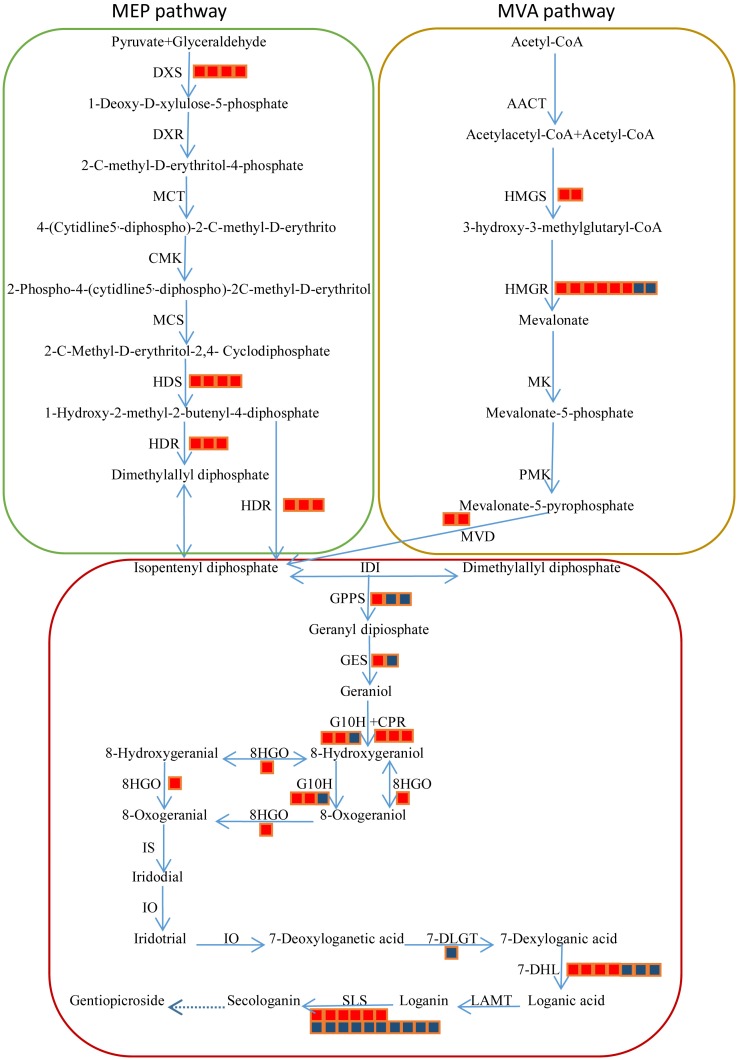
DEGs involved in gentiopicroside biosynthesis pathway in M5 samples compared with C samples. DXS, 1-deoxy-D-xylulose-5-phosphate synthase; DXR, deoxylulose 5-phosphate reductoisomerase; MCT, 2-C-methyl-D-erythritol 4-phosphate cytidylyltransferase; CMK, 4-(cytidine -5’-diphospho)- 2-C-methyl-D- erythritol kinase; MCS, 2-C-methyl-D-erythritol-2,4- cyclodiphosphate synthase; HDS, 1-hydroxy-2-methyl-2-(E)-butenyl 4-diphosphate synthase; HDR, 1-hydroxy-2-methyl -2-(E)-butenyl 4-diphosphate reductase; IDI, isopentenyl diphosphate isomerase; AACT, acetyl-CoA C-acetyltransferase; HMGS, 3-hydroxy-3-methylglutaryl-coenzyme A synthase; HMGR, 3-hydroxy-3- methylglutaryl-coenzyme A reductase; MK, mevalonate kinase; PMK, 5-phosphomevelonate kinase; MVD, mevalonate-5-pyrophosphate decarboxylase; GPPS, geranyl diphosphate synthase; GES, geraniol synthase; G10H, geraniol 8-oxidase/geraniol 10-hydroxylase; CPR, cytochrome P450 reductase; 8/10HGO, 8-hydroxygeraniol oxidoreductase; IS, iridoid synthase; IO, iridoid oxidase; 7-DLGT, 7-deoxyloganetic acid glucosyl transferase; 7-DLH, 7-deoxyloganic acid hydroxylase; LAMT, loganic acid O-methyltransferase; SLS, secologanin synthase. Red squares, putative encoding genes are up-regulated; blue squares, putative encoding genes are down-regulated.

Our RNA-seq data indicated that 243 unigenes, including 59 DEGs, are members of the gentiopicroside biosynthesis pathway. Among them, 114 unigenes, including 23 DEGs, are involved in the IPP pathway while 129 unigenes, including 36 DEGs, function in secoiridoid biosynthesis (Table 4). Although we did not find here that the latter pathway was significantly enriched, we did note that the former pathway is (Fig 6). Expression of 21 DEGs related to the IPP pathway was up-regulated in plants treated with MeJA. Among the 36 DEGs involved in iridoid biosynthesis, 18 were up-regulated and 18 were down-regulated. These results demonstrated that applying MeJA could significantly increase gentiopicroside biosynthesis by up-regulating the expression of genes related to the IPP pathway, but not to the secoiridoid biosynthesis pathway.

**Table 4:** Putative genes that encode enzymes involved in gentiopicroside biosynthesis and DEGs for which expression is altered in response to MeJA treatment.

Gene	No.^a^	Up^b^	Down^c^
***MEP pathway***			
1-deoxy-D-xylulose-5-phosphate synthase (DXS)	22	4	-
1-deoxy-D-xylulose-5-phosphate reductoisomerase (DXR)	7	-	-
2-C-methyl-D-erythritol 4-phosphate cytidylyltransferase (MCT)	3	-	-
4-diphosphocytidyl-2-C-methyl-D-erythritol kinase (CMK)	2	-	-
2-C-methyl-D-erythritol-2,4-cyclodiphosphate synthase (MCS)	4	-	-
4-hydroxy-3-methylbut-2-enyl diphosphate synthase (HDS)	17	4	-
4-hydroxy-3-methylbut-2-enyldiphosphate reductase (HDR)	5	3	-
***MVA pathway***			
Acetyl-CoA acetyltransferase (AACT)	13	-	-
Hydroxymethylglutaryl-CoA synthase (HMGS)	5	2	-
3-hydroxy-3-methylglutaryl-coenzyme A reductase (HMGR)	24	6	2
Mevalonate kinase (MK)	3	-	-
Phosphomevalonate kinase (PMK)	7	-	-
mevalonate-5-pyrophosphate decarboxylase (MVD)	2	2	-
***Iridoid biosynthesis***			
Isopentenyl-diphosphate delta-isomerase (IDI)	6	-	-
Geranyl diphosphate synthase (GPPS)	15	1	2
Geraniol synthase (GES)	4	1	1
Geraniol 8-oxidase/geraniol 10-hydroxylase (G10H)	16	2	1
8/10-hydroxygeraniol oxidoreductase (8/10HGO)	3	1	-
Iridoid synthase (IS)	2	-	-
Iridoid oxidase (IO)	1	-	-
7-deoxyloganetic acid glucosyltransferase (7-DLGT)	18	-	1
7-deoxyloganic acid hydroxylase (7-DLH)	9	4	3
loganic acid O-methyltransferase (LAMT)	-	-	-
Secologanin synthase (SLS)	32	6	10
Cytochrome P450 reductase (CPR)	23	3	-
Total	243	39	20

^a^ number of unique sequences encoding putative enzymes.

^b^ number of up-regulated DEGs.

^c^ number of down-regulated DEGs (false discovery rate, or FDR <0.001).

### DEGs involved in glycolysis

**Fig 7:**
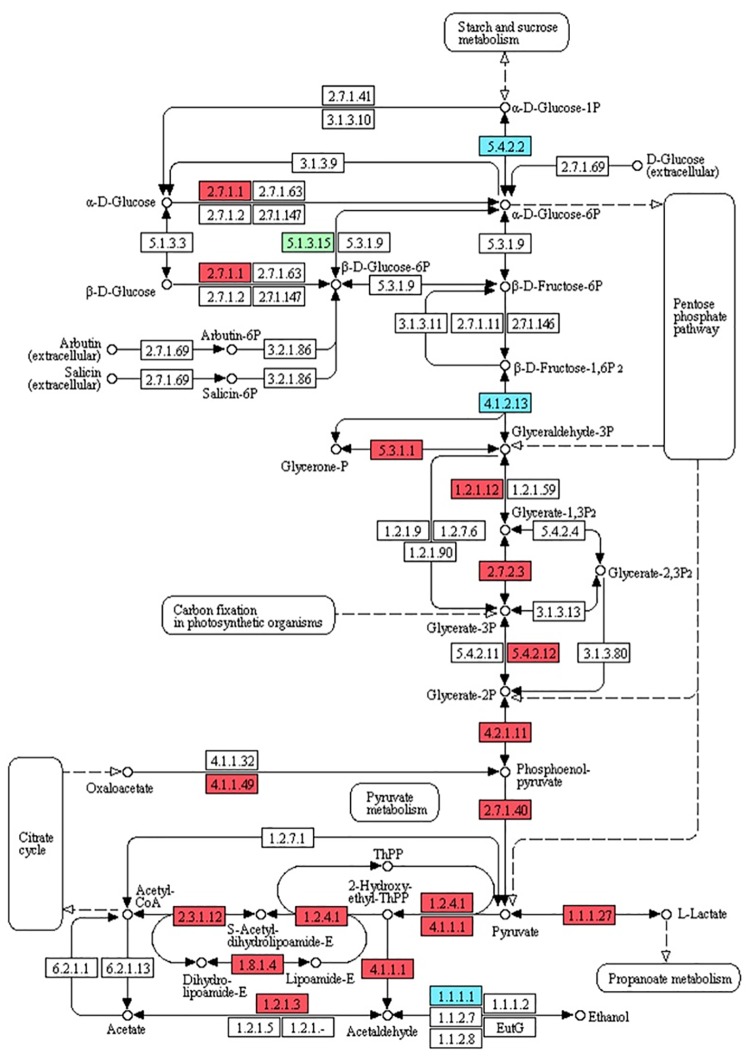
DEGs involved in glycolysis in M5 samples compared with C samples. 5.4.2.2, phosphoglucomutase; 2.7.1.1, hexokinase; 5.1.3.15, glucose-6-phosphate 1-epimerase; 4.1.2.13, fructose-bisphosphate aldolase; 5.3.1.1, triosephosphate isomerase; 1.2.1.12, glyceraldehyde 3-phosphate dehydrogenase; 2.7.2.3, phosphoglycerate kinase; 5.4.2.12, 2,3-bisphosphoglycerate- independent phosphoglycerate mutase; 4.2.1.11, enolase; 2.7.1.40, pyruvate kinase; 4.1.1.49, phosphoenolpyruvate carboxykinase; 1.2.4.1, pyruvate dehydrogenase E1 component alpha subunit; 4.1.1.1, pyruvate decarboxylase; 2.3.1.12, dihydrolipoamide acetyltransferase; 1.1.1.27, L-lactate dehydrogenase; 1.8.1.4, dihydrolipoamide dehydrogenase; 1.2.1.3, aldehyde dehydrogenase; 1.1.1.1, alcohol dehydrogenase. Green box, putative encoding gene is down-regulated; red boxes, putative encoding genes are up-regulated; blue boxes, some putative encoding genes are up-regulated while others are down-regulated.

Glucose is broken down by glycolysis to produce acetyl coenzyme A (acetyl-CoA) as the direct precursor in the MVP pathway, as well as glyceraldehyde-3-phosphate and pyruvate, which are precursors in the MEP pathway [33]. We found that the glycolysis pathway was significantly enriched in M5 samples when compared with C samples (Fig 7) and 52 of 57 related DEGs were up-regulated (S5 Table). For example, in that pathway, many putative genes encoding hexokinase, fructose-bisphosphate aldolase, triosephosphate isomerase, glyceraldehyde 3-phosphate dehydrogenase, phosphoglycerate kinase, gpmI, enolase, pyruvate kinase, phosphoenolpyruvate carboxykinase, pyruvate dehydrogenase E1 component alpha subunit, pyruvate decarboxylase, dihydrolipoamide acetyltransferase, L-lactate dehydrogenase, dihydrolipoamide dehydrogenase, and aldehyde dehydrogenase were significantly up-regulated by MeJA treatment (Fig 7, S5 Table).

### DEGs associated with hormone signaling components

We observed that treatment with MeJA triggered the enrichment of signal transduction pathways for GA, ET, SA, and ABA. In addition to MYC2 and JAZs in the JA signaling pathway, transcripts of signaling components such as the gibberellin receptor GID1, DELLA, and phytochrome-interacting factor 4 for GA; ethylene receptor ETR, ethylene-insensitive protein 3, and ethylene-responsive transcription factor 1 (ERF1) for ET; pathogenesis-related protein 1 (PR1) for SA; and protein phosphatase 2C and serine/threonine-protein kinase for ABA were greatly accumulated in response to MeJA (S6 Table). We were interested to find that expression of the PR1 mRNA was increased by 576-fold.

### DEGs associated with TFs

We identified 164 DEGs that responded to MeJA elicitation, including 131 that were up-regulated and 33 that were down-regulated (S7 Table). They were largely represented by TF families that influence secondary metabolism and stress responses, e.g., the ERF superfamily (32 members), bHLH superfamily (27 members), WRKY superfamily (19 members), and myeloblastosis (MYB) superfamily (15 members). Transcript levels of the five most up-regulated genes increased by 105.3- to 212.1-fold in M5 samples, with three of them putatively encoding members of the ERF superfamily. In contrast, transcript levels of the five most strongly down-regulated genes decreased by 23.2- to 60.9-fold in response to MeJA, with three of them putatively encoding members of the bHLH superfamily.

### Validation of gene expression by qRT-PCR

**Fig 8:**
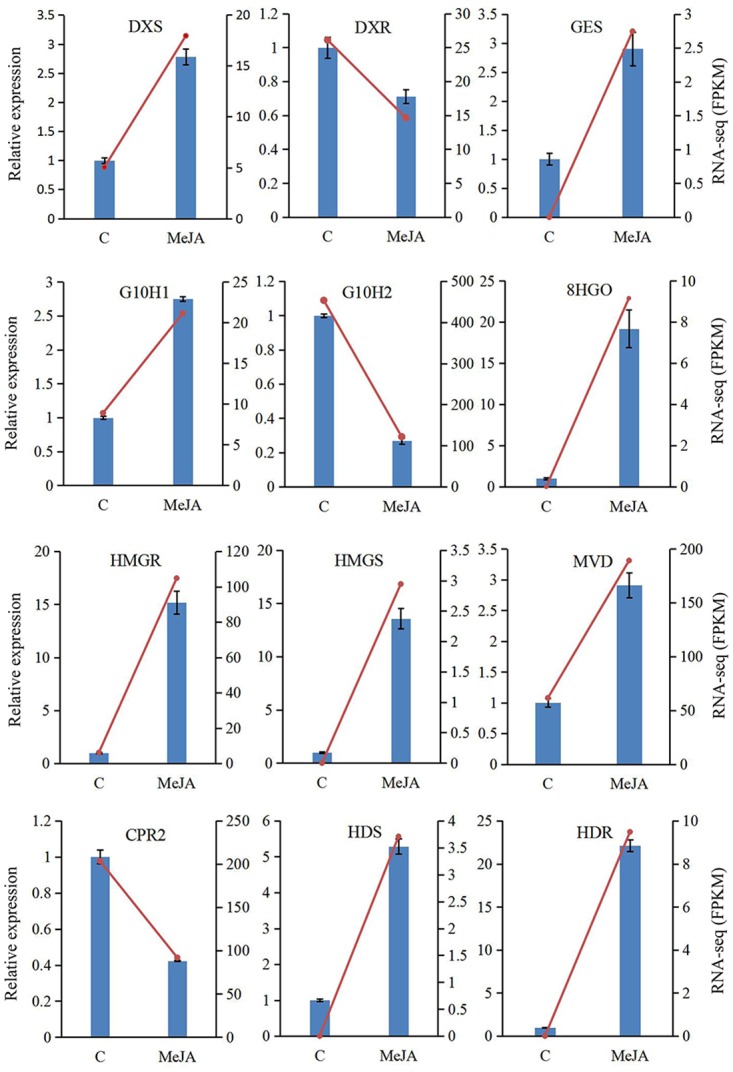
qRT-PCR validation of 12 unigenes involved in gentiopicroside biosynthesis in *G*. *macrophylla* seedlings. DXS, 1-deoxy-D-xylulose-5-phosphate synthase; DXR, deoxylulose 5-phosphate reductoisomerase; GES, geraniol synthase; G10H, geraniol 8-oxidase/geraniol 10-hydroxylase; 8/10HGO, 8/10-hydroxygeraniol oxidoreductase; HMGR, 3-hydroxy-3-methylglutaryl-coenzyme A reductase; HMGS, 3-hydroxy-3-methylglutaryl-coenzyme A synthase; MVD, mevalonate-5- pyrophosphate decarboxylase; CPR, cytochrome P450 reductase; HDS, 1-hydroxy-2-methyl-2- (E)-butenyl 4-diphosphate synthase; HDR, 1-hydroxy-2-methyl-2-(E)-butenyl 4-diphosphate reductase.

We used qRT-PCR analysis to validate the important DEGs obtained from our assembled transcriptome as well as from the expression profiles revealed by RNA-Seq data. This examination entailed 12 unigenes involved in the biosynthesis of IPP (DXS, DXR, HDS, HDR, HDS, HMGR, and MVD) and secoiridoids (GES, G10H, and 8/10HGO) (Fig 8). Our results suggested that the assembled transcripts were reliable and that the designed primer pairs were suitable for subsequent expression experiments. Based on the 2^-ΔΔCt^ method [31], relative expression levels of the selected unigenes were calculated and compared between M5 and C samples. The expression patterns detected by qRT-PCR were consistent with those from the RNA-Seq data. Overall, the qRT-PCR analysis confirmed that the unigenes obtained from the assembled transcriptome were trustworthy and the profiles were credible.

## Discussion

Exogenous MeJA is believed to be a primary regulator of pathways for JA biosynthesis and signaling in plants. This compound has been studied extensively in *Solanum lycopersicum*, *Arabidopsis thaliana*, and *Taxus chinensis* [6,34,35]. Jasmonates are plant-specific signaling molecules that activate several defense mechanisms, inducing a massive reprogramming of gene expression [36]. The basic helix—loop—helix (bHLH) TF MYC2 is a central regulator in JA signaling cascades, including those leading to the biosynthesis of several classes of specialized metabolites [37]. In the absence of JA, MYC2 action is repressed by jasmonate ZIM-domain-containing (JAZ) proteins by forming repressor complexes with a group of other proteins such as Novel Interactor of JAZ (NINJA) and TOPLESS [38,39]. This JA elicitation leads to JAZ degradation by the SCFCOI1—ubiquitin—proteasome pathway, thereby releasing MYC2 from the repressor complex. In the present transcriptome pool, there are 7 putative unigenes encoding MYC2 and 18 putative genes encoding JAZs based on BLASTs performed against public databases (S1 Table). We found that the expression of all DEGs encoding putative MYC2 and JAZ were up-regulated in MeJA-treated seedlings (Table 3). The JA signaling mechanism also oscillates through a negative feedback loop involving MYC2 and JAZ proteins, in which JAZ blocks MYC2 activity at the protein level while MYC2 transcriptionally induces JAZ expression [34]. Our results clearly confirmed that exogenous application of MeJA can regulate the pathways for JA biosynthesis and signaling in *G*. *macrophylla*.

The derivation of gentiopicroside from secologanin entails 10 enzymatic conversions, starting from IPP (Fig 6). *Catharanthus roseus* has been used to investigate several genes that encode key enzymes in those pathways [15,40]. Briefly, IPP is produced via the plastidial MEP or cytosolic MVA pathway (Fig 6). However, the biosynthetic route from secologanin to gentiopicroside has been entirely unknown to date. We previously identified 114 putative unigenes involved in secoiridoid biosynthesis in our transcriptome library [41]. In the present database, 40 putative unigenes encoding iridoid synthase (IS), iridoid oxidase (IO), 7-deoxyloganetic acid glucosyltransferase (7-DLGT), and 7-deoxyloganic acid hydroxylase (7-DLH) were identified for the first time in *G*. *macrophylla*.

The glycolysis pathway (Fig 7) not only plays a crucial role in energy generation but also provides carbon building blocks for the biosynthesis of gentiopicroside and other organic constituents of secoiridoid [42,43]. In particular, glucose is broken down by glycolysis to produce acetyl coenzyme A (acetyl-CoA) as the direct precursor in the MVP pathway, as well as glyceraldehyde-3-phosphate and pyruvate, which are precursors in the MEP pathway [33]. In the present study we found that the glycolysis pathway was significantly enriched in M5 samples when compared with C samples (Fig 7) and up-regulation of numerous enzyme genes led to an elevated rate of flux and replenished the precursors consumed in IPP biosynthesis. Research on the rubber tree (*Hevea brasiliensis*) has suggested that such rapid acceleration of the glycolysis pathway in supplying precursors for the production of IPP and natural rubber, rather than rubber biosynthesis per se, is responsible for ethylene (ET) stimulation of latex yields [44]. Here, we noted that the high expression of enzyme genes in both the glycolysis pathway and the IPP biosynthesis pathway contributed to greatly increased gentiopicroside concentrations in response to MeJA.

Hormone responses are generally the result of interactions and crosstalk among multiple pathways [45]. For example, JA signaling may function by interacting with other major plant hormones, such as ET, gibberellin (GA), alicylic acid (SA), abscisic acid (ABA), brassinosteroid, auxin, and cytokinin. Signaling crosstalk between SA and JA results in complementary action in mediating endophyte-induced accumulations of secondary metabolites [46,47]. Moreover, under stress conditions, ABA interacts with the SA/JA pathways [48]. Although some plant hormones, e.g., ABA, ET, and SA, induce the production of bioactive compounds [44,49], it is unclear how JA interacts and coordinates with other hormones with regard to this stimulation. Our results showed that treatment with MeJA triggered the enrichment of signal transduction pathways for GA, ET, SA, and ABA. One explanation for this crosstalk is that IPP is also the precursor of GA and ABA biosynthesis and that MeJA also elicits a rise in IPP production.

Transcription factors play important roles in controlling many biological processes in a cell or organism by modulating gene expression [50]. Many TFs help regulate the biosynthesis and accumulation of secondary metabolites [51], and also have crucial roles in crosstalk between hormone signalling pathways [52]. For example, CrWRKY1, a member of the WRKY family from *Catharanthus roseus*, can positively regulate TIA biosynthesis [53]. The phenylpropanoid pathway in different plant organs and tissues of higher plants is under the control of specific R2R3-MYB TFs and bHLH families [54]. We identified many putative transcription factors differentially expressed between C samples and M5 samples (S7 Table), including the ERF superfamily, bHLH superfamily, WRKY superfamily, and myeloblastosis (MYB) superfamily. Although it is still unknown how these genes function in parallel with MeJA applications to enhance the production of gentiopicroside, our data provide a valuable resource for discovering candidate genes related to the complex regulatory networks involved in that response.

## Conclusion

Although MeJA is a ubiquitous and conserved elicitor of plant secondary metabolism, the degree to which metabolic pathways are stimulated is species-specific. We verified here that MeJA applications effectively enhance the production of gentiopicroside in *Gentiana macrophylla*. Our RNA-Seq analysis of MeJA-related transcriptional changes indicated that 5206 genes are differentially expressed. Transcriptome analysis revealed increased expression of genes in the α-lenolenic acid degradation pathway that produces abundant JA and quickly activates the holistic JA pathway in those seedlings. Rapid acceleration of the glycolysis pathway that supplies precursors for IPP biosynthesis and up-regulates the expression of enzyme genes in that IPP pathway are probably most responsible for MeJA stimulation of gentiopicroside synthesis. Furthermore, many genes encoding various TFs, e.g., ERF, bHLH, MYB, and WRKY, also respond in plants exposed to MeJA.

The results from this research improve our understanding of how MeJA applications alter the production of gentiopicroside in *G*. *macrophylla* seedlings. Our data will provide a massive genetic resource for further investigation of gentiopicroside biosynthesis and will lay the foundation for genetic engineering to boost yields of this compound in such a valuable medical plant.

## Supporting Information

S1 Table Functional annotations of unigenes summarized from alignments to the NR, Swiss-Prot, Pfam, KOG, KEGG, GO, and COG databases. (XLS)

S2 Table The 20 most-represented pathways in the *Gentiana macrophylla* transcriptome. (DOCX)

S3 Table Genes differentially expressed between C samples and M5 samples. (XLS)

S4 Table The 20 most up-regulated and down-regulated genes between C and M5 samples. (XLS)

S5 Table Genes involved in the glycolysis pathway that are differentially expressed between C samples and M5 samples. (DOCX)

S6 Table Genes involved in hormone signaling components that are differentially expressed between C samples and M5 samples. (DOCX)

S7 Table Putative transcription factors differentially expressed between C samples and M5 samples. (XLSX)

S8 Table Primer sequences used for qRT-PCR. (DOCX)
