# Supplementary material for: Transcriptional Responses and Gentiopicroside Biosynthesis in Methyl Jasmonate-Treated Gentiana macrophylla Seedlings
Source: PLoS One. 2016 Nov 16;11(11):e0166493. doi: 10.1371/journal.pone.0166493 (PMC5112864; doi:10.1371/journal.pone.0166493)
Supplement: S2 Table — (DOCX) [file pone.0166493.s002.docx]

**Table S2** The 20 most-represented pathways in the *Gentiana macrophylla* transcriptome

| Metabolism pathway | KEGG ORTHOLOGY (KO) entry | Number of unigenes （percent） |
| --- | --- | --- |
| Carbon metabolism | ko01200 | 872 (4.47%) |
| Ribosome | ko03010 | 772 (3.96%) |
| Biosynthesis of amino acids | ko01230 | 743 (3.81%) |
| Protein processing in endoplasmic reticulum | ko04141 | 697 (3.57%) |
| Spliceosome | ko03040 | 563 (2.89%) |
| RNA transport | ko03013 | 515 (2.64%) |
| Starch and sucrose metabolism | ko00500 | 497 (2.55%) |
| Oxidative phosphorylation | ko00190 | 440 (2.26%) |
| Plant hormone signal transduction | ko04075 | 426 (2.18%) |
| Endocytosis | ko04144 | 386 (1.98%) |
| Glycolysis / Gluconeogenesis | ko00010 | 375 (1.92%) |
| Plant-pathogen interaction | ko04626 | 370 (1.90%) |
| Ubiquitin mediated proteolysis | ko04120 | 356 (1.83%) |
| Phenylpropanoid biosynthesis | ko00940 | 349 (1.79%) |
| mRNA surveillance pathway | ko03015 | 342 (1.75%) |
| Amino sugar and nucleotide sugar metabolism | ko00520 | 329 (1.69%) |
| Purine metabolism | ko00230 | 320 (1.64%) |
| RNA degradation | ko03018 | 310 (1.59%) |
| Pyruvate metabolism | ko00620 | 309 (1.58%) |
| Carbon fixation in photosynthetic organisms | ko00710 | 277 (1.42%) |
