# Supplementary material for: Transcriptional Responses and Gentiopicroside Biosynthesis in Methyl Jasmonate-Treated Gentiana macrophylla Seedlings
Source: PLoS One. 2016 Nov 16;11(11):e0166493. doi: 10.1371/journal.pone.0166493 (PMC5112864; doi:10.1371/journal.pone.0166493)
Supplement: S5 Table — (DOCX) [file pone.0166493.s005.docx]

**Table S5** Genes involved in the glycolysis pathway that are differentially expressed between C samples and M5 samples.

| **Enzyme (ID)** | **KEGG orthology** | **Sequence ID** | **log2FC** | **Fold changes** |
| --- | --- | --- | --- | --- |
| Phosphoglucomutase (pgm)  (EC:[5.4.2.2](http://www.kegg.jp/dbget-bin/www_bget?ec:5.4.2.2)) | K01835 | c47293.graph_c1 | 5.55 | 46.85 |
|  |  | c85014.graph_c0 | 4.98 | 31.56 |
|  |  | c19646.graph_c0 | -1.88 | 3.68 |
| Hexokinase (HK)  (EC:[2.7.1.1](http://www.kegg.jp/dbget-bin/www_bget?ec:2.7.1.1)) | K00844 | c26075.graph_c0 | 5.14 | 35.26 |
|  |  | c78327.graph_c0 | 1.72 | 3.29 |
|  |  | c88125.graph_c0 | 4.50 | 22.63 |
| glucose-6-phosphate 1-epimerase (EC:[5.1.3.15](http://www.kegg.jp/dbget-bin/www_bget?ec:5.1.3.15)) | K01792 | c75106.graph_c0 | -2.30 | 4.92 |
| fructose-bisphosphate aldolase (ALDO)  (EC:[4.1.2.13](http://www.kegg.jp/dbget-bin/www_bget?ec:4.1.2.13)) | K01623 | c18081.graph_c0 | 4.58 | 23.92 |
|  |  | c44352.graph_c0 | 4.72 | 26.35 |
|  |  | c57332.graph_c0 | 5.19 | 36.50 |
|  |  | c58020.graph_c0 | 4.02 | 16.22 |
|  |  | c58104.graph_c0 | 4.65 | 25.11 |
|  |  | c65321.graph_c0 | 5.83 | 56.89 |
|  |  | c68313.graph_c0 | 4.33 | 20.11 |
|  |  | c84915.graph_c0 | 4.02 | 16.22 |
|  |  | c85299.graph_c0 | 4.23 | 18.77 |
|  |  | c83620.graph_c0 | -1.80 | 3.48 |
| triosephosphate isomerase (TIM) (EC:[5.3.1.1](http://www.kegg.jp/dbget-bin/www_bget?ec:5.3.1.1)) | K01803 | c17474.graph_c0 | 3.58 | 11.96 |
|  |  | c58983.graph_c0 | 4.72 | 26.35 |
| glyceraldehyde 3-phosphate dehydrogenase (GAPDH) (EC:[1.2.1.12](http://www.kegg.jp/dbget-bin/www_bget?ec:1.2.1.12)) | K00134 | c50140.graph_c0 | 2.77 | 6.82 |
|  |  | c64560.graph_c0 | 4.13 | 17.51 |
|  |  | c84673.graph_c0 | 4.12 | 17.39 |
|  |  | c84777.graph_c0 | 4.42 | 21.41 |
| phosphoglycerate kinase (PGK) (EC:[2.7.2.3](http://www.kegg.jp/dbget-bin/www_bget?ec:2.7.2.3)) | K00927 | c48755.graph_c0 | 5.70 | 51.98 |
|  |  | c72307.graph_c0 | 4.92 | 30.27 |
| 2,3-bisphosphoglycerate-independent phosphoglycerate mutase (gpmI) (EC:[5.4.2.12](http://www.kegg.jp/dbget-bin/www_bget?ec:5.4.2.12)) | K15633 | c65567.graph_c0 | 4.58 | 23.92 |
|  |  | c85173.graph_c0 | 4.92 | 30.27 |
|  |  | c85330.graph_c0 | 4.23 | 18.77 |
| Enolase (ENO)  (EC:[4.2.1.11](http://www.kegg.jp/dbget-bin/www_bget?ec:4.2.1.11)) | K01689 | c19582.graph_c0 | 5.29 | 39.12 |
|  |  | c64492.graph_c0 | 4.65 | 25.11 |
| pyruvate kinase (PK)  (EC:[2.7.1.40](http://www.kegg.jp/dbget-bin/www_bget?ec:2.7.1.40)) | K00873 | c64649.graph_c0 | 4.86 | 29.04 |
|  |  | c65891.graph_c0 | 4.65 | 25.11 |
|  |  | c87292.graph_c0 | 4.02 | 16.22 |
|  |  | c88058.graph_c0 | 4.33 | 20.11 |
| phosphoenolpyruvate carboxykinase (ATP) (EC:[4.1.1.49](http://www.kegg.jp/dbget-bin/www_bget?ec:4.1.1.49)) | K01610 | c54933.graph_c0 | 5.93 | 60.97 |
|  |  | c84563.graph_c0 | 5.34 | 40.50 |
| pyruvate dehydrogenase E1 component alpha subunit (PDHA)(EC:[1.2.4.1](http://www.kegg.jp/dbget-bin/www_bget?ec:1.2.4.1)) | K00161 | c15978.graph_c0 | 4.42 | 21.41 |
| pyruvate decarboxylase (PDC) (EC:[4.1.1.1](http://www.kegg.jp/dbget-bin/www_bget?ec:4.1.1.1)) | K01568 | c26435.graph_c0 | 8.84 | 458.251 |
|  |  | c86234.graph_c0 | 4.86 | 29.04 |
| dihydrolipoamide acetyltransferase (DLAT) (EC:[2.3.1.12](http://www.kegg.jp/dbget-bin/www_bget?ec:2.3.1.12)) | K00627 | c39490.graph_c0 | 4.86 | 29.041 |
| L-lactate dehydrogenase (LDH) (EC:[1.1.1.27](http://www.kegg.jp/dbget-bin/www_bget?ec:1.1.1.27)) | K00016 | c66171.graph_c0 | 6.01 | 64.45 |
| dihydrolipoamide dehydrogenase (DLD) (EC:[1.8.1.4](http://www.kegg.jp/dbget-bin/www_bget?ec:1.8.1.4)) | K00382 | c17883.graph_c0 | 5.38 | 41.64 |
|  |  | c76759.graph_c0 | 4.86 | 29.04 |
| aldehyde dehydrogenase (ALDH) (EC:[1.2.1.3](http://www.kegg.jp/dbget-bin/www_bget?ec:1.2.1.3)) | K00128 | c18929.graph_c0 | 6.72 | 105.42 |
|  |  | c65839.graph_c0 | 4.02 | 16.22 |
|  |  | c75711.graph_c0 | 5.09 | 34.06 |
|  |  | c76192.graph_c0 | 2.63 | 6.19 |
|  | K14085 | c58092.graph_c0 | 4.50 | 22.63 |
| alcohol dehydrogenase (ADH) (EC:[1.1.1.1](http://www.kegg.jp/dbget-bin/www_bget?ec:1.1.1.1)) | K00121 | c62311.graph_c0 | 5.66 | 50.56 |
|  |  | c63123.graph_c0 | 4.92 | 30.27 |
|  |  | c63362.graph_c0 | 5.96 | 62.25 |
|  |  | c64754.graph_c0 | 6.55 | 93.70 |
|  |  | c80928.graph_c0 | 2.31 | 4.96 |
|  |  | c84889.graph_c0 | 6.12 | 69.55 |
|  |  | c68677.graph_c0 | -3.46 | 11.00 |
|  |  | c70904.graph_c1 | -3.63 | 12.38 |
|  | K18857 | c48600.graph_c0 | 4.02 | 16.22 |
