# Supplementary material for: Transcriptional Responses and Gentiopicroside Biosynthesis in Methyl Jasmonate-Treated Gentiana macrophylla Seedlings
Source: PLoS One. 2016 Nov 16;11(11):e0166493. doi: 10.1371/journal.pone.0166493 (PMC5112864; doi:10.1371/journal.pone.0166493)
Supplement: S6 Table — (DOCX) [file pone.0166493.s006.docx]

**Table S6** Genes involved in hormone signaling components that are differentially expressed between C samples and M5 samples

| Enzyme (ID) | KEGG orthology | Sequence ID | log2FC | Fold changes |
| --- | --- | --- | --- | --- |
| gibberellin receptor (GID1) | K14493 | c53241.graph_c0 | 6.49 | 89.88 |
|  |  | c65420.graph_c0 | 4.23 | 18.77 |
| DELLA protein | K14494 | c59670.graph_c0 | 4.13 | 17.51 |
| phytochrome-interacting factor 4 (PIF4) | K16189 | c67430.graph_c0 | 4.50 | 22.63 |
| ethylene receptor (ETR) | K14509 | c87134.graph_c0 | 5.29 | 39.12 |
| ethylene-insensitive protein 3 (EIN3) | K14514 | c37273.graph_c0 | 4.65 | 25.11 |
|  |  | c60882.graph_c0 | 4.13 | 17.51 |
|  |  | c78607.graph_c0 | 5.73 | 53.08 |
| ethylene-responsive transcription factor 1 (ERF1) | K14516 | c29190.graph_c0 | 2.63 | 6.19 |
| pathogenesis-related protein 1 (PR1) | K13449 | c56414.graph_c0 | 9.17 | 576.03 |
| protein phosphatase 2C （PP2C） | K14497 | c17967.graph_c0 | 5.29 | 39.12 |
|  |  | c41003.graph_c0 | 6.10 | 68.59 |
|  |  | c74624.graph_c0 | 1.92 | 3.78 |
|  |  | c85551.graph_c0 | 6.12 | 69.55 |
| serine/threonine-protein kinase （SNRK2） | K14494 | c59670.graph_c0 | 4.13 | 17.51 |
| abscisic acid receptor (PYL) | K14496 | c55754.graph_c0 | 4.58 | 23.92 |
|  |  | c49868.graph_c0 | -2.57 | 5.94 |
| protein brassinosteroid insensitive 2 (BIN2) | K14502 | c17994.graph_c0 | 3.35 | 10.20 |
| cyclin D3 (CYCD3) | K14505 | c80073.graph_c0 | -1.58 | 2.99 |
| auxin influx carrier (AUX1) | K13946 | c71608.graph_c0 | -1.51 | 2.85 |
| transport inhibitor response 1 (TIR1) | K14485 | c20327.graph_c0 | 4.92 | 30.27 |
| auxin-responsive protein (IAA) | K14484 | c16338.graph_c0 | 5.14 | 35.26 |
|  |  | c18687.graph_c0 | 6.65 | 100.43 |
|  |  | c18758.graph_c0 | 7.45 | 174.85 |
|  |  | c19002.graph_c0 | 4.92 | 30.27 |
|  |  | c28245.graph_c0 | 6.96 | 124.50 |
|  |  | c44782.graph_c0 | 5.66 | 50.56 |
|  |  | c69590.graph_c0 | 5.04 | 32.90 |
|  |  | c78914.graph_c0 | 4.79 | 27.67 |
|  |  | c84059.graph_c0 | 7.29 | 156.50 |
|  |  | c84138.graph_c0 | 7.44 | 173.65 |
|  |  | c84150.graph_c0 | 5.51 | 45.57 |
|  |  | c84423.graph_c0 | 6.12 | 69.55 |
|  |  | c84726.graph_c0 | 5.77 | 54.57 |
|  |  | c87936.graph_c0 | 4.02 | 16.22 |
| auxin responsive (GH3) | K14487 | c24920.graph_c0 | 4.42 | 21.41 |
|  |  | c70492.graph_c0 | 2.14 | 4.41 |
|  |  | c84396.graph_c0 | 7.81 | 224.41 |
|  |  | c27323.graph_c0 | -6.30 | 78.79 |
| SAUR | K14488 | c16620.graph_c1 | 4.65 | 25.11 |
|  |  | c70882.graph_c0 | 5.96 | 62.25 |
|  |  | c80305.graph_c2 | 4.65 | 25.11 |
|  |  | c47026.graph_c0 | -4.39 | 20.97 |
|  |  | c61420.graph_c0 | -4.14 | 17.63 |
|  |  | c65375.graph_c0 | -2.21 | 4.63 |
|  |  | c67807.graph_c0 | -5.05 | 33.13 |
|  |  | c73476.graph_c0 | -2.50 | 5.66 |
|  |  | c76973.graph_c0 | -2.16 | 4.47 |
| histidine-containing phosphotransfer (AHP) | K14490 | c18751.graph_c0 | 3.06 | 0.12 |
|  |  | c62613.graph_c0 | -4.38 | 20.82 |
| two-component response regulator ARR-B | K14491 | c73270.graph_c0 | 4.02 | 16.22 |
|  |  | c86735.graph_c0 | 5.24 | 37.79 |
| two-component response regulator ARR-A | K14492 | c45808.graph_c0 | -2.09 | 4.26 |
|  |  | c54139.graph_c0 | -2.24 | 4.72 |
| JAZ protein | K13464 | c37174.graph_c0 | 4.27 | 19.29 |
|  |  | c38617.graph_c0 | 4.72 | 26.35 |
|  |  | c48886.graph_c0 | 5.09 | 34.06 |
|  |  | c63000.graph_c0 | 2.77 | 6.82 |
|  |  | c65729.graph_c0 | 4.02 | 16.22 |
|  |  | c66883.graph_c0 | 1.88 | 3.68 |
|  |  | c71121.graph_c0 | 4.86 | 29.04 |
|  |  | c86508.graph_c0 | 4.42 | 21.41 |
| MYC2 | K13422 | c77634.graph_c0 | 2.11 | 4.32 |
|  |  | c78484.graph_c0 | 1.75 | 3.36 |
