# Supplementary material for: Transcriptional Responses and Gentiopicroside Biosynthesis in Methyl Jasmonate-Treated Gentiana macrophylla Seedlings
Source: PLoS One. 2016 Nov 16;11(11):e0166493. doi: 10.1371/journal.pone.0166493 (PMC5112864; doi:10.1371/journal.pone.0166493)
Supplement: S8 Table — (DOCX) [file pone.0166493.s008.docx]

**Table S8:** Primer sequences used for qRT-PCR

| Genes | Unigene number | Sequence |
| --- | --- | --- |
| *GmSAND1* | Unigene80256 | 5'-TTCATGGTGATTCTCCAGC-3’  5'-TTCAAGGAAGATGACAACC-3’ |
| *GmCPR2* | Unigene68944 | 5'- ACCGCACTTTCCAAATACGCA-3’  5'- CCGAGGAGCAACTGAGGCAAA-3’ |
| *GmDXR* | Unigene59623 | 5'- TGGGATGGTCCAAAGCCGA-3’  5'- CCTTGTTAGCCAGGGCAATGTC-3’ |
| *GmG10H1* | Unigene75247 | 5'-AAACGCTCTCTACGCCCACGA-3’  5'-TACCGACATCCACCGCCTCA-3’ |
| *GmG10H2* | Unigene66392 | 5'- CCAAGTAGTCTCATCACGCCCG-3’  5'- CGAGCAAGAAGTGGAGGTGTGTG-3’ |
| *GmDXS* | Unigene30005 | 5'-CCATAGCGGCGTGTATAGCA-3’  5'-TACGGAGAAAGGACGAGGGT-3’ |
| *GmHDS* | Unigene68377 | 5'-TTGGTTATGTTGGCGGTGCT-3’  5'-TCACTCTTCAGTTGGAGGTTCA-3’ |
| *GmIDS* | Unigene72244 | 5'-CTATGGTGACAGGACCTTTTGG-3’  5'-TGGAGGATGGAACTCAAGCA-3’ |
| *GmHMGS* | Unigene85156 | 5'-TATGTTCATCAGTGACCGTTGG-3’  5'-TGTGACAAACCAGGACATTAGC-3’ |
| *GmHMGR* | Unigene59493 | 5'-GCGTCGGTTATTTATCTTGTGGG-3’  5'-CTTAGACGGCTGAGATGGAACCT-3’ |
| *GmMVD* | Unigene70938 | 5'-CTGCTCTATGTTGTATGAGGGGAC-3’  5'-TATGGCGGATTTGTGAAGTGG-3’ |
| *GmGES* | Unigene47392 | 5'-ACTTGTGGAAAGAGCTGAATGG-3’  5'-GGAGAAAGAAGTCACAAAGGGTCA-3’ |
| *Gm8HGO* | Unigene62311 | 5'-AGGAGAGGTTTTTGGAATAACGGAG-3’  5'-CCACTTGTCTCAAATCCAGCA-3’ |
